# Prompt Antiviral Action of Pulmonary CD8+ T_RM_ Cells Is Mediated by Rapid IFN-γ Induction and Its Downstream ISGs in the Lung

**DOI:** 10.3389/fimmu.2022.839455

**Published:** 2022-02-22

**Authors:** Lang Jiang, Lu Liu, Miaomiao Zhang, Linxia Zhang, Cuisong Zhu, Qian He, Lilin Ye, Chen Zhao, Zejun Li, Jianqing Xu, Xiaoyan Zhang

**Affiliations:** ^1^ Institute of Clinical Science & Shanghai Key Laboratory of Organ Transplantation, Zhongshan Hospital, Institutes of Biomedical Sciences, Fudan University, Shanghai, China; ^2^ Shanghai Public Health Clinical Center, Fudan University, Shanghai, China; ^3^ Obstetrics & Gynecology Hospital, Institutes of Biomedical Sciences, Fudan University, Shanghai, China; ^4^ Institute of Immunology, Third Military Medical University, Chongqing, China; ^5^ Shanghai Veterinary Research Institute, Chinese Academic of Agricultural Sciences, Shanghai, China

**Keywords:** influenza, lung-resident memory CD8 T cells, IFN-γ, interferon-induced genes, inflammatory cytokines

## Abstract

Growing lines of evidence supported the importance of CD8+ lung tissue resident memory T (T_RM_) cells in protection against respiratory viruses, exemplified by influenza A virus. However, the underlying *in vivo* mechanism remains largely undetermined. Here, we used mouse infection models to dissect *in vivo* cross-protective activity of lung CD8+ T_RM_ cells. By simultaneously interrogating transcriptional dynamics in lung CD8+ T_RM_ cells and surrounding tissues during the early course of infection, we demonstrated that lung CD8+ T_RM_ cells react to antigen re-exposure within hours, manifested by IFN-γ upregulation, and a tissue-wide interferon-stimulated gene (ISG) program is subsequently elicited. Using antibody-mediated IFN-γ neutralization and IFN-γ receptor knockout mice, we could show that the induction of several important antiviral ISGs required IFN-γ signaling, so did the suppression of key inflammatory cytokines. Interestingly, there were also examples of ISGs unaffected in the absence of IFN-γ activity. Collectively, focusing on *in situ* characterization of lung CD8+ T_RM_ cells during very early stage of infection, a critical period of host antiviral defense that has been poorly investigated, our studies highlight that these cells, once triggered by antigen re-exposure, are programmed to produce IFN-γ expeditiously to promote a lung-wide antiviral response for effective virus control.

## Introduction

Influenza A viruses (IAVs) continued to be a serious public threat and vaccination is considered as the major, if not the only, means for its control at the population level (1). The major currently used IAV vaccine works by inducing neutralizing antibody against the head domain of viral hemagglutinin protein (HA), which is responsible for virus entry by binding to sialic acid receptors on cell surface (2, 3). However, the head domain is highly plastic with significant sequence diversification between different IAVs, even within the same subtype; consequently, the vaccine efficacy is often confined to the virus strains that are used for vaccine preparation, accounting for the necessity of annual vaccination (2–5). The virus composition of seasonal flu vaccine is determined based on surveillance data of currently circulating viruses, which was used to predict the virus strains with the greatest likelihood of circulating during the coming season. Thus, the variation in prediction accuracy has been translated into year-to-year variation in protective efficacy of IAV vaccine. The emergency of mismatched epidemic and pandemic influenza variants driven by antigenic drift or shift posed further challenge for neutralizing an antibody-based IAV vaccine (2). A possible solution to this conundrum is exploration of more conserved HA stalk as immunogen for vaccine design.

T cell-mediated cellular response represents the other arm of adaptive immunity. The importance of CD8+ T-cell response in anti-influenza immunity was evidenced by analyses of patients infected with pandemic H1N1 virus, demonstrating a direct correlation between virus-specific CD8+ T cells and cross-protection against symptomatic influenza (6). Our study on the victims of the 2013 H7N9 outbreak further revealed that an earlier potent virus-specific CD8+ T-cell response is a critical contributor to a faster recovery (7). Compared to humoral immunity, cellular immunity is more likely to mediate cross-protection by targeting viral internal proteins, which are more conserved than the surface proteins—the primary target of neutralizing antibodies.

Memory T cells consist of three primary subsets: central memory cells (T_CM_), effector memory cells (T_EM_), and more recently identified tissue-resident cells (T_RM_). Unlike T_CM_ and T_EM_ cells, which circulate and account for body-wide protection, T_RM_ cells persist in the various non-lymphoid tissues where the prior encounter with antigen/immunogen occurs and in turn acts as a local sentinel sensing and protecting the residing tissue from invasion of the same pathogen or those possessing the same antigen (8). T_RM_ is characterized by a distinct transcriptional core profile and differential expression of surface markers exemplified by CD69 and CD103. On the other side, T_RM_ from different tissues may diverge from each other in surface marker(s), longevity, and functionality, implying tissue-specific adaptation. For example, liver T_RM_ cells express LFA-1 instead of CD103 (9) while intestinal T_RM_ cells contain both CD103-positive and CD103-negative subpopulations (10). Skin T_RM_ cells persist in the epidermal niche through *in situ* self-renewal, in line with the provision of a long-lived immune protection independently of enduring local antigen stimulation (11–14). Contrastingly, the number of lung T_RM_ declined over time, and its maintenance may require continued replenishment from circulating memory T-cell pools (15–18).

Although a number of murine studies have supported the importance of lung CD8+ T_RM_ cells in memory recall response against IAV (19–24) and suggested its elicitation as a critical measurement of a vaccine effective in cross-subtype protection, the protective mechanism of lung T_RM_ cells is not yet fully understood. CD8+ T_RM_ cells might execute their antiviral function through directly killing the infected cells (25) or establishing an antiviral milieu through secretion of antiviral cytokines represented by IFN-γ (26). The second mechanism was proposed on the basis of functional characterization of mouse female reproductive tract (FRT) and skin T_RM_ cells, positing that activation by reencountering cognate antigen triggers T_RM_ to release IFN-γ, which induces expression of ISGs to establish a tissue-wide antiviral response (26, 27). Owing to the broad antiviral activities of some of the IFN-γ-induced ISGs, this response is endowed with capacity to be cross-protective. In addition, IFN-γ may cooperate with other cytokines released by T_RM_ to orchestrate maturation of dendritic cells (DCs) and natural killer (NK) cells, as well as recruitment of circulating memory T and B cell to fortify local antiviral immunity (27). Given the phenotype diversity among different T_RM_ cells, the generality of the IFN-γ paradigm remains to be vindicated. In the case of lung T_RM_ cells, only a few published reports explored the mechanistic aspect of their antiviral functions. McMaster et al. initially showed by an intratracheal transfer approach that airway T_RM_ cells were sufficient for mounting effective protection against influenza virus infection in an antigen-specific manner (20). The same study also characterized the *ex vivo* responses of airway T_RM_ cells to antigen stimulation and identified rapid production of IFN-γ with little cell proliferation as the major event, which was subsequently substantiated by the finding that viral control conveyed by transfer of airway IFN-γ-deficient T_RM_ cells was less effective than that achieved by wild-type T_RM_ cells. According to our knowledge, there has been no published report on *in situ* dynamics of lung T_RM_ cells during an effective protective response.

Here, we used mouse infection models to assess the protective potential of lung CD8+ T_RM_ cells and interrogate their working mechanism by a combinatory approach. In particular, we probed the early action of activated lung CD8+ T_RM_ cells by using microarray to analyze transcriptional changes in both these cells and the lung over the course of early phase of infection. This paralleled analysis, combined with following experimental corroborations, established rapid production of IFN-γ to induce a tissue-wide expression of ISGs while suppressing inflammation as the major, but not the only, mechanism utilized by lung T_RM_ cells to exert their protective functions. Together, our work shed new insights into the biology of lung CD8+ T_RM_ cells and thereby provided new mechanistic basis for their optimal exploitation to protect against respiratory viral infection as well as other diseases.

## Materials and Methods

### Mice

C57BL/6J (B6) mice were originally purchased from the Jackson Laboratory; P14 CD45.1 transgenic mouse was a gift from Dr. Lilin Ye (Army Medical University, Chongqin); we obtained the IFN-γ receptor 1 (Ifngr1) knockout mice, which were generated by CRISPR/Cas9-mediated genome engineering, from Cyagen Biosciences (https://www.cyagen.com/cn/zh-cn/sperm-bank-live/15979). The mice were propagated and maintained under specific pathogen-free (SPF) conditions in the animal facility at Shanghai Public Health Clinical Center (SPHCC). Six-week-old female mice were used throughout the study with approval from the Institutional Animal Care and Use Committee (IACUC) of SHPCC.

### Viruses and Infection Models

The A/Shanghai/4664T/2013 (H7N9) virus was from Bio-Safety level 3 laboratory at SHPCC; the A/Chicken/Shanghai/F/98 (H9N2) virus was kindly provided by Dr. Zejun Li (Shanghai Academy of Agricultural Sciences). Recombinant Puerto Rico/8/34 (PR8, H1N1) virus expressing gp33 and LCMV Armstrong strain were kindly provided by Dr. Lilin Ye (Army Medical University, Chongqing). All the used influenza viruses were propagated in 9-day-old embryonated eggs and the 50% tissue culture infectious dose (TCID50) titers of virus stocks were determined in MDCK cells in accordance with the Reed–Muench method; the LCMV Armstrong was grown in BHK21 cells and the titers of virus stock were determined in VERO cells utilizing immune focus assay with anti-LCMV antibody (Clone: M104, Abcam). For infection, virus was adjusted with phosphate-buffered saline (PBS) to a final volume of 50 μl on ice. Two sequential models were employed in the study: in one model, naïve C57BL/6J mice were first intranasally (i.n.) infected with 150 TCID50 of H9N2 and, after an interval as indicated, i.n. exposed to 1×10^4^ TCID50 of H7N9 virus; in the other model, 5×10^4^ P14 CD8+ T cells were isolated from P14 CD45.1 transgenic mouse and seeded into naïve C57BL/6J mice, which were i.n. primed with 50 TCID50 of PR8/PR8-gp33 1 day later and after waiting for 30 days, subjected to intranasal challenge with 2×10^5^ PFU of LCMV Armstrong. The procedures related to H9N2 and H7N9 viruses were conducted in Bio-Safety Level 3 Laboratory while the handlings of other viruses were performed in Bio-Safety Level 2 Laboratory.

### Microarray Analysis

For preparation of RNA samples, infected lung tissues were pulverized with a mortar and pestle under liquid nitrogen and total RNA were then purified by Direct-zol RNA MiniPrep Kit (Zymo Research, Irvine, CA, USA) following extraction with RNAzol (Molecular Research Center Inc., USA). The quality of RNA was ensured by measurement on an Agilent Bioanalyzer 2100 using RNA NanoChip. RNA preparations from three lungs isolated at the same sample time point were pooled together for microarray analysis. For transcriptomic analysis of lung TRM cells derived from adoptively transferred P14 cells, lung bronchoalveolar lavage (BAL) of three mice was harvested at each assay time point, from which CD45.1+CD69+CD11a- T_RM_ cells were sorted through flow cytometry before pooling together (a total of 7,000–20,000 cells) for RNA extraction. Microarray analyses were performed with Affymetrix Clariom S Mouse Gene Expression Chip. Data were log2-transformed and normalized to median. GO and pathway enrichment analyses were carried out using DAVID Bioinformatics Resources. The gene expressions of IFNs and ISGs were further analyzed using Multiple Experiment Viewer (MeV).

### Flow Cytometry Analysis

The following fluorescence-activated cell sorting (FACS) antibodies were purchased from BD Biosciences, BioLegend, or eBioscience: PB/AF700/Percp-cy5.5 anti-CD3 (145-2C11), PB/FITC/AF488/Percp-cy5.5/APC anti-CD8a (RPA-T8), FITC/PE/APC/Percp-cy5.5 anti-CD45.1 (A20), PE anti-CD69 (H1.2F3), AmCyan/BV510/Percp-cy5.5 anti-CD103 (2E7), BV785 anti-PD1 (29F.1A12), PE/PE-cy7 anti-IFN-γ (XMG1.2), AF488 anti-CD11a (M17/4), APC/PE-cy7 anti-CD62L (MEL-14), FITC anti-CD44 (IM7), BV421/BV510 anti-CXCR3 (CXCR3-173), BV605 anti-KLRG1 (2F1), and BV421 anti-CD127 (A7R34). Tetramer APC NP366–374/H-2D(b) and Live/dead-AmCyan/BV510 were purchased respectively from MEDICAL & BIOLOGICAL LABORATORIES CO., LTD. and Invitrogen. Cell staining was performed essentially as described previously (28) and samples were acquired on the BD LSRFortessa or sorted by BD FACSaria. Data were then analyzed by FlowJo V10.4.2 (TreeStar) or BD FACSDiva™.

### Cytometric Bead Array Assay

Infected lung was lavaged with 1 ml of PBS to collect BAL fluid. The concentrations of cytokines in BAL fluid were determined by bead-based multiplex LEGENDplex™ analysis (LEGENDplex™ Mouse Anti-Virus Response Panel, 13-plex; BioLegend) according to the manufacturer’s protocol. Cytokines measured included IFNα, IFNβ, IFNγ, TNFα, IL-1β, IL-6, IL-10, IL-12p70, GM-CSF, CCL2, CCL5, CXCL1, and CXCL10. The samples were acquired using a BD LSRFortessa™ flow cytometer and data analyses were performed with Legendplex V8.0 software (BioLegend); cytokine concentrations were expressed in pg/ml.

### CD8 T-Cell Depletion and IFN-γ Neutralization

Anti-CD8, anti-IFN-γ, and isotype-matched control antibodies were purchased from Bio X Cell. For CD8+ T-cell depletion, 200 μg of antibody in 100 µl of PBS was delivered into each animal intranasally or intraperitoneally at 1 day before infection and repeated at day 1 and day 3 post infection. IFN-γ neutralization was performed by intranasal inoculation of 200 μg of antibody in 50 μl of PBS at indicated time points after infection.

### RNAscope *In Situ* Hybridization

Mice were deeply anesthetized with single intraperitoneal injection of 1% phenobarbital sodium in PBS. Lungs were rapidly removed, snap frozen in the Optimal Cutting Temperature (OCT) embedding media with liquid nitrogen, and then stored at −80°C until sectioned. Tissues blocks were cut by a CM1950 cryostat (Leica, Wetzlar, Germany) into 14-μm-thick sections, which were subsequently mounted on positively charged slides. The slides were dried for 10 min at −20°C before being stored at −80°C until further processing. RNAscope analyses were performed by using RNAscope^®^ Multiplex Fluorescent Kit (Advanced Cell Diagnostics, Inc, Newark, CA), following the manufacturer’s instructions. In brief, the sections were baked at 60°C for 30 min, fixed with 10% NBF (neutral buffered formalin solution) at 4°C for 1 h, and then dehydrated with increasing concentrations of ethanol (50%, 70%, and 100% ethanol), followed by protease treatment before incubation with an RNAscope probe specific to mouse IFN-γ mRNA. The sections were imaged on TissueFAXS Confocal 200 (Tissue Gnostics) and analyzed by Strata Quest 6.0X software.

### Quantitative RT-PCR

First-strand complementary DNAs (cDNAs) were synthesized from 1 μg of each RNA sample by Reverse Transcription System and quantified by real-time PCR with SYBR Green PCR Mix (Promega) on an Applied Biosystems 7300 RT-PCR cycler. The relative gene expressions were calculated by the delta-delta Ct method with β-Actin used as the reference gene. Absolute quantification of viral load was obtained by extrapolating the Ct value against a copy number standard curve. The primers are shown in the **supplementary table**.

### Statistical Analysis

All statistical analyses were performed using GraphPad Prism 8. Comparison of two groups was performed using Mann–Whitney Unpaired *t*-test. One-way or two-way analysis of variance (ANOVA) was used to determine the difference between more than two groups, and survival was analyzed by the Kaplan–Meier method with the log-rank test. A *p*-value <0.05 was considered statistically significant.

## Results

### Development of Two Mouse Infection Models for Assessment of Cross-Protection Against Pulmonary Virus Infection Mediated by Memory Response

**Figure 1:**
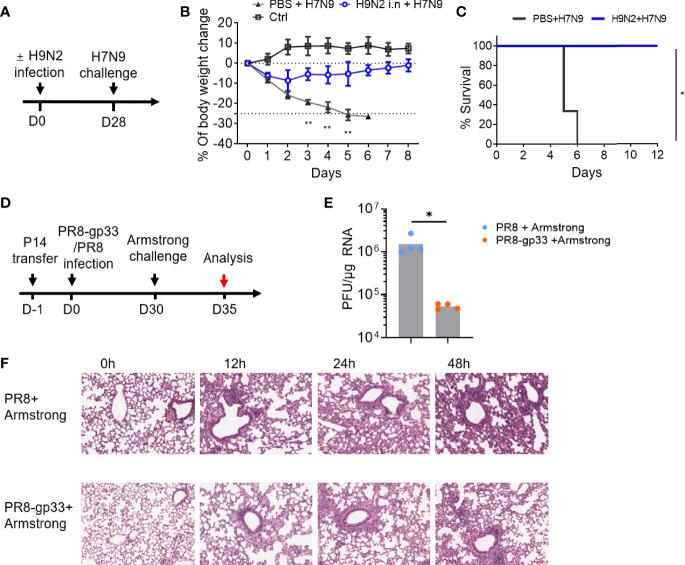
Mouse infection models demonstrated the cross-protectivity of T-cell memory response to virus infection. **(A–C)** Exploration of harmless H9N2 virus as a live vaccine against pathogenic H7N9 virus. The sequential intranasal infections of naïve mice with H9N2 and H7N9 viruses were performed according to a 4-week schedule **(A)**. Following H7N9 challenge, the mice were daily monitored for weight changes with the control (ctrl) group representing the animal group only receiving PBS **(B)** and survival **(C)**. **(D–F)** An experimental approach combining adoptive transfer and sequential virus infections to demonstrate cross protectivity of memory CD8+ T cell *via* cross-recognizing a single epitope. The experimental scheme was illustrated in **(D)**. In brief, mice were adoptively transferred with 5 ×10^4^ naïve P14 CD8+ T cells, then i.n. infected with sublethal dose of PR8 or PR8-gp33 as the prime, and 30 days later challenged with LCMV Armstrong at 2 × 10^5^ of plaque‐forming units (PFU). Shown in **(E)** was lung viral load determination at day 5 post LCMV Armstrong challenge, and in **(F)** were representative images of H&E-stained lung sections sampled at the indicated time points post challenge. Scale bar (red): 100 μm. Data are representative of two or three independent experiments with *n* = 3 **(B, C)**. Mean ± SEM **(B)**. *p < 0.05, **p < 0.01; Log-rank test **(C)**; Mann–Whitney *U* test **(E)**.

Emerging avian-origin H7N9 virus is considered as a severe threat to human health due to its high mortality and pandemic-causing potential. During our course of exploration of vaccination strategy against H7N9 infection, we found in a mouse model that prior infection with low pathogenic, non-disease-causing H9N2 strain could confer a cross-protection against heterologous H7N9 challenge. This finding was made in a study where naïve C57BL/6 mice were first intranasally (i.n.) inoculated with PBS or H9N2 virus and 4 weeks later challenged with a lethal H7N9 infection (**Figure 1A**). In contrast to the PBS-primed control group, which suffered a continuous weight loss, the H9N2-primed mice exhibited only moderate weight loss at the first day post infection (dpi), thereafter stabilizing and progressing toward a full recovery that was attained at 8 dpi (**Figure 1B**). Consequently, all animals in the PBS-primed group had died by 6 dpi, whereas the H9N2-primed group showed 100% survival at the end of the 12-day observation period (**Figure 1C**). Given that the used H9N2 and H7N9 viruses share six internal genes while differing in genes encoding hemagglutinin (HA) and neuraminidase (NA) surface proteins, we speculated that the observed cross-subtype protection is most likely mediated by memory T-cell response targeting conserved internal epitopes.

To facilitate dissection of mechanism mediating the cross-protection of lung CD8+ T_RM_ cells, we developed a second experimental model combining adoptive cell transfer and sequential infections of two viruses that are of different types while sharing an immunodominant CD8+ T-cell epitope. In this model, as schematically illustrated in **Figure 1D**, CD45.1+ CD8+ T cells were isolated from P14 mice, which carries an engineered T-cell receptor specifically recognizing the CD8(H-2Db) gp33 epitope of glycoprotein of lymphocytic choriomeningitis virus (LCMV) (amino acids 33–41; KAVYNFATC), and adoptively transferred into naïve C57BL/6 mice; the recipient mice were then primed with unlethal dose (50 TCID50) of A/PR/8 H1N1 (PR8) virus or a recombinant PR8 virus with LCMV gp33 epitope inserted into the neuraminidase glycoprotein (PR8-gp33) and awaited for 30 days for memory induction before challenging with the second infection, 2 × 10^5^ plaque-forming units (PFU) of LCMV Armstrong. The lung viral load at 5 dpi after the LCMV Armstrong challenge was approximately 25-fold lower in PR8-gp33-primed mice than that in PR8-primed mice (**Figure 1E**), in line with improved lung histopathology during the first 48 h after infection, as assessed by H & E staining (**Figure 1F**). Thus, as underscored by the second model, memory CD8 + T cells deposited by primary influenza infection could act alone to protect against subsequent heterologous virus infection through engagement of a single shared epitope.

### Antigen-Specific Lung CD8+ T_RM_ Cells Have Potent Antiviral Activities

**Figure 2:**
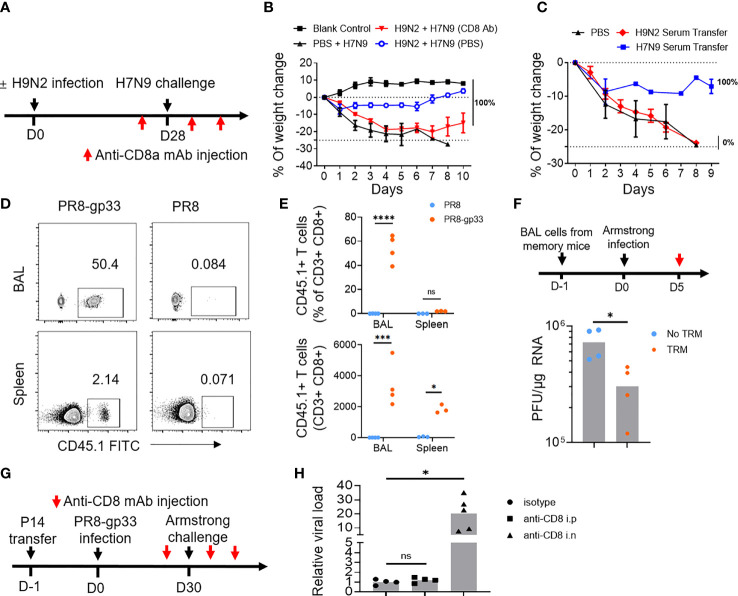
Lung CD8+ T_RM_ cells have high potential to afford cross-protein against virus infection. **(A, B)** Mice primed with PBS or H9N2 infection were challenged with a lethal dose of H7N9 virus following the schedule described in **Figure 1A**, except that intraperitoneal injection of anti-CD8a or control antibody was applied at 1 day prior to H7N9 challenge as well as day 1 and day 3 after the challenge **(A)**. H7N9 infection-induced weight changes were daily monitored **(B)**. **(C)** Mice received transfusion of serum from H9N2- or H7N9-infected mice and were subsequently challenged with a lethal dose of H7N9. The survival rate was determined daily after the challenge. **(D–F)** Mice were seeded with P14 cells by adoptive transfer and infected with PR8-gp33 or PR8 virus. Thirty days later, the abundance of P14 cells in the bronchoalveolar lavage (BAL) and spleen of resulting memory mice were analyzed by flow cytometry, with representative flow cytometry plots shown in **(D)**, and pooled absolute and percent (among total CD8+ population) enumeration data shown in **(E)**. The BAL fluids were harvested from memory mice (*n* = 5) and i.n. transferred into naïve mice, which were then challenged with i.n. infection of LCMV Armstrong and 5 days later subjected to lung viral load determination **(F)**. **(G, H)** Memory mice were developed the same as in **(D, E)**, followed by the LCMV Armstrong challenge, along with anti-CD8 or isotype control antibody being injected either intranasally (i.n.) or intraperitoneally (i.p.) both at 1 day before and at 1 day and 3 days after the challenge **(G)**. **(H)** Lung viral loads were determined at day 5 after the LCMV Armstrong challenge. Data are representative of two or three independent experiments with *n* = 3 **(B, C)**. *p < 0.05, ***p < 0.001, ****p < 0.0001; ns, not significant; two-way ANOVA **(E)**; Mann–Whitney *U* test **(F**, **H)**.

Next, we investigated the immune mechanism responsible for the H9N2 priming-induced cross-protection against H7N9. To this end, we first assessed the contribution of circulating CD8+ T cells by antibody-mediated depletion (**Figure S1A**). Depletion of circulating CD8+ T cells by intraperitoneal administration of an anti-CD8 antibody compromised but not abolished (still resulting in 100% survival) the cross-protection (**Figures 2A, B**). We also observed that transferring of H9N2-primed serum to naïve mice did not result in significant enhancement in protection against subsequent H7N9 challenge as seen with H7N9 primed serum, which is supposed to enrich for anti-H7 neutralizing antibodies (**Figure 2C**). These results together substantiated the notion that cellular response involving both systematic and resident CD8+ T cells, rather than antibody response, mainly accounts for the H9N2-mediated protection against subsequent H7N9 challenge. Following this, we analyzed the phenotype of influenza-specific memory CD8+ T cells in the bronchoalveolar lavage (BAL) at 28 dpi after H9N2 priming by flow cytometry. The surface marker profiling of NP tetramer-positive BAL CD8+ T cells was characterized by high expression of PD-1, CXCR3, CD44, CD69, and low expression of CCR7, CD25, KLRG-1, and CD127, with only a small portion of cells expressing CD103 (**Figures S2A, B**). This profiling was consistent with lung T_RM_ phenotype previously reported (19, 29).

We further employed the second mouse model to dissect the contribution of systematic and lung resident memory CD8+ T cells to the cross-protective immunity conferred by prior influenza infection. We first interrogated memory P14 cells by flow cytometry at 30 days after the PR8 or PR8-gp33 priming (just before the LCMV Armstrong challenge). The PR8-gp33-primed mice showed a remarkable enrichment of P14 memory cells in the BAL, accounting for 50% of the total CD8+ population with a total number of approximately 4,000. There was also detectable accumulation of P14 cells in the spleen, albeit lower in frequency and number as compared to those in BAL. In contrast, P14 cells were barely detectable in either compartment in PR8-primed mice (**Figures 2D, E**). The BAL P14 cells were characterized by high expression of CD69, PD-1 and CXCR3 and low expression of CD103 (**Figures S2C, D**), again conforming to the known airway T_RM_ phenotype.

We then used two approaches to appraise the antiviral activity of PR8-gp33-induced lung memory P14 cells. In the first approach, we transferred BAL from PR8-gp33- or PR8-primed lung to naïve mice, followed by infection with LCMV Armstrong virus and determination of virus load at 5 dpi. The mice receiving PR8-gp33-primed BAL showed significantly better virus control than those receiving PR8-primed BAL, consistent with the idea that PR8-gp33 but not PR8 priming can induce the formation of lung memory P14 cell population, which is recalled by subsequent infection of LCMV Armstrong virus through recognition of the gp33 epitope and consequently afford protection (**Figure 2F**). In the second approach, we depleted circulating CD8+ T cells and lung CD8+ T_RM_ cells respectively by i.p. and i.n. injection of anti-CD8 antibody (**Figure S1B**), and compared the effects on the control of subsequent LCMV Armstrong challenge. An isotype-matched IgG mAb was used as the control (**Figure 2G**). Depletion of lung CD8+ T_RM_ cells led to approximately 30-fold increase in viral load, in sharp contrast to no observable effect shown by blockade of circulating CD8+ memory T cells (**Figure 2H**). These results, together with the aforementioned data obtained with the H9N2-primed protection against H7N9, confirmed and extended earlier findings that virus-specific lung T_RM_ cells, particularly those residing in airway, can effectively provide cross-protection in an antigen-dependent manner.

### Lung T_RM_ Cells Rapidly Produce IFN-γ in Response to Antigen Re-Exposure, Directing an Early IFN-Stimulated Gene expression Program in the Lung

Previous studies on FRT-resident and skin-resident T_RM_ cells suggested that a major function of activated T_RM_ cells is triggering a state of pathogen alert in its residence by secreting IFN-γ (26, 27). A similar mechanism has been proposed for lung T_RM_ cells with supporting evidence mainly from previous work by McMaster et al., showing that isolated airway T_RM_ cells were capable of rapidly producing IFN-γ while exhibiting very limited cytolytic activity when stimulated with cognate antigen (20). However, there is lack of *in vivo* characterization of lung T_RM_ activity at the early stage of infection.

**Figure 3:**
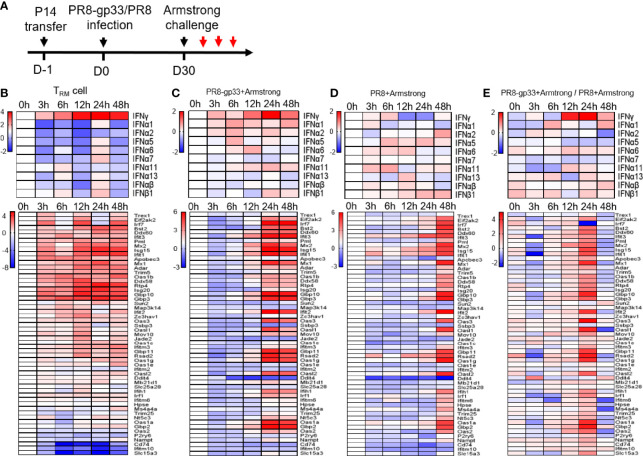
Activated lung T_RM_ cells rapidly upregulate IFN-γ, associated with induction of ISG expression inside themselves and in the lung. Shown in **(A)** is the experimental scheme. Naïve mice received P14 cells by adoptive transfer and were then infected with a sublethal dose of PR8-gp33 virus or control PR8 virus, 30 days later challenged with LCMV Armstrong. The lung and BAL fluid were harvested from animals at 0 h, 3 h, 6 h, 12 h, 24 h, and 48 h after the challenge. P14 cells were isolated from the BAL fluid through flow cytometry. Microarray analyses were performed on RNAs pooled from three lungs or P14 cells isolated from three BAL samples. The revealed dynamic expression pattern of different IFNs and downstream ISGs were respectively shown as the upper and lower panel for lung CD8+ T_RM_ cells **(B)** and lungs from the two treatment groups **(C, D)**. Shown in **(E)** is the heatmap constructed by the ratio of mRNA values obtained in **(C, D)**.

To explore the mechanistic aspects of the protective action of lung CD8+ T_RM_ cells, we utilized the second infection model throughout the rest of this study. We first sought to gain a better understanding of the *in vivo* response of lung CD8+ T_RM_ cells to antigen re-exposure. We thus isolated lung P14 T_RM_ cells from BAL at various time points within 0–48 h post LCMV Armstrong infection; RNA samples were prepared and subjected to microarray profiling (**Figure 3A**). Among the detected IFNs, IFN-γ emerges as the only IFN displaying increased mRNA levels during the entire assay period with clear upregulation being observed as early as 3 hpi, which continued to rise until peaking at 24 hpi and then decreased moderately at 48 hpi. The majority, if not all, of type I IFN genes also saw an increase in mRNA level, but it was significantly delayed as compared to IFN-γ, mostly evident at 24 hpi (**Figure 3B**). We further analyzed the mRNA dynamics of ISGs downstream of IFN and observed a substantial divergence in expression patterns. One group of ISGs, as represented by several well-known anti-influenza ISGs (Gbp3, Ifit3, Isg15, and Mx1/2), showed a kinetic pattern of mRNA expression largely resembling that of IFN-γ mRNA, suggesting that they were likely to be directly regulated by IFN-γ in an autocrine manner. These ISGs were also the most highly induced. On the opposite side, there were also ISGs showing only little to moderate induction throughout the assessment period, implicating that they are not downstream targets of IFN-γ-signaling or their activation by IFN-γ in lung CD8+ T_RM_ cells is suppressed by other signaling(s). Between the two groups is a group characterized by delayed kinetics of induction; we conceived that those ISGs could be downstream of IFN-γ (albeit requiring a stronger IFN-γ stimulation) or of the later produced IFN-α/β. Thus, lung T_RM_ cells are transcriptionally poised to upregulate IFN-γ rapidly and continually upon sensing the viral antigen, and they might be also committed to induce expression of type I IFNs at specific later time point(s) as the infection continues. On the other hand, their exhibition of strong induction of antiviral ISGs with a temporal pattern paralleling that of IFN-γ suggested that the activity of these cells might be also regulated by self-produced IFN-γ.

Next, we examined the effects of lung CD8+ T_RM_ cell activation on the surrounding tissues by comparing mRNA expression profiles of PR8-gp33-primed vs. PR8-primed lungs collected at the same time points as described above for lung CD8+ T_RM_ cell isolation. The gene expression pattern of IFN-γ in PR8-gp33-primed lungs was highly similar to that observed with lung CD8+ T_RM_ cells, featuring a continuous rise between 3 and 24 hpi and a sign of decline at 48 hpi (**Figure 3C**). Such similarity, together with the fact that PR8-primed lungs showed little induction of IFN-γ mRNA at 12 hpi and 24 hpi relative to 0 hpi (**Figure 3D**), was consistent with the notion that lung CD8+ T_RM_ cells are the major source of IFN-γ in the lung at the early phase of infection. The analysis of ISGs mRNA expression provided further evidence: for PR8-gp33-primed lungs, a wide array of ISGs were strongly induced by 24 hpi and remained at similar levels at 48 hpi, with the induction of a large portion of them being already discernable at 12 hpi (**Figure 3C**); for PR8-primed lungs, robust induction of a broad spectrum of ISGs was also observed, but it was delayed until 48 hpi (**Figure 3D**). The concomitant earlier induction of IFN-γ and ISGs in PR8-gp33-primed lungs relative to PR8-primed lungs was better visualized in the heatmap constructed using the gene expression ratio between the two lung types (**Figure 3E**). Two differences in the expression profiling of ISGs should be noted between PR8-gp33-primed lungs and lung CD8+ T_RM_ cells: (1) For the group of ISGs showing high induction in both contexts, lung T_RM_ cells displayed faster kinetics, which could be explained by the fact that IFN-γ secreted by lung T_RM_ cells can be immediately captured by these cells while requiring time to reach lung cells at a distance; (2) the spectrum of highly induced ISGs was broader in PR8-gp33-primed lungs than lung CD8+ T_RM_ cells, indicating that activated lung CD8+ T_RM_ cells elicit ISG expression program in the lung mainly through acting on lung cells other than on themselves.

**Figure 4:**
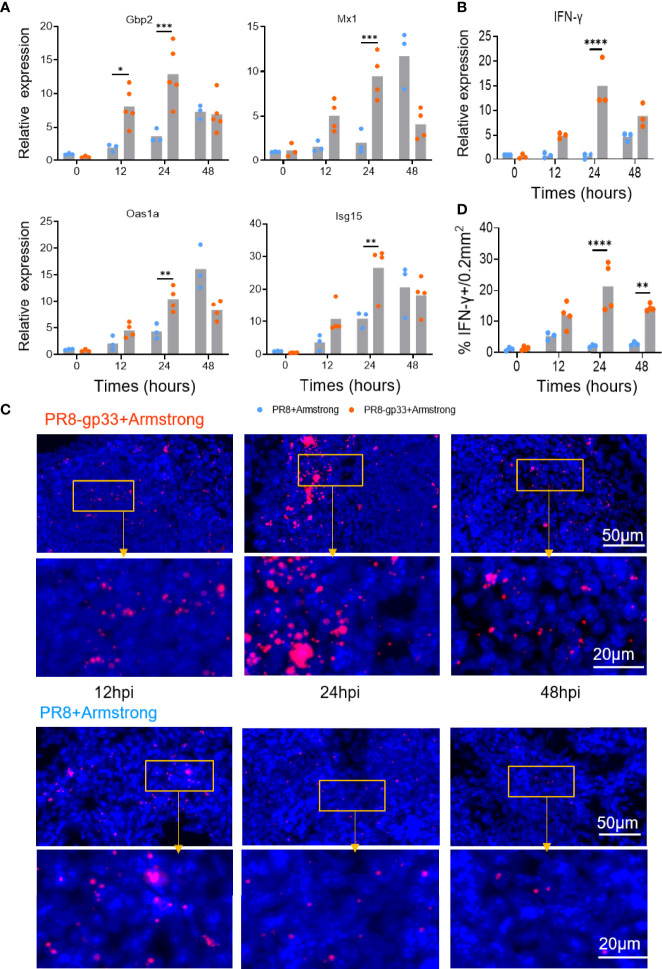
Validation of microarray data by RT-PCR and RNAscope *in situ* hybridization. **(A, B)** Animals were treated following the same scheme shown in **Figure 3**, and lungs were collected at the indicated time points after LCMV Armstrong challenge for quantitative RT-PCR analysis of mRNA expression of four representative ISGs **(A)** and lung IFN-γ **(B)**. **(C, D)** Dynamics of lung IFN-γ mRNA determined by RNAscope *in situ* hybridization, with representative lung sections shown in **(C)** and quantification data shown in **(D)**. Data are representative of two or three independent experiments. *p < 0.05, **p < 0.01, ***p < 0.001, ****p < 0.0001; two-way ANOVA **(A, B, D)**.

We subsequently verified the above microarray data by using quantitative RT-PCR to measure mRNA levels of IFN-γ and four representative antiviral ISGs including Gbp2, Mx1, Oas1a, and Isg15. The measurements of PR8-gp33-primed lung samples showed a course of induction shared by the five mRNAs that was overall consistent with that revealed by microarray: the mRNA induction was readily detectable at 12 hpi, peaking at 24 hpi before declining to some extent at 48 hpi (except for Mx1, whose induction peaked at 48 hpi). By comparison, with PR8 priming, IFN-γ mRNA saw no substantial elevation throughout the 48-h observation period while the degree of the induction of individual ISG mRNA was significantly lower at 12–24 hpi but became comparable or even higher at 48 hpi (**Figures 4A, B**). We further analyzed the expression of IFN-γ mRNA *in situ* using RNAscope hybridization assay. The enumeration of fluorescent (IFN-γ mRNA positive) dots indicated 24 hpi as the time point when the presence of lung CD8+ T_RM_ cells resulted in maximal induction of IFN-γ at the transcriptional level, corroborating the microarray and RT-PCR analyses (**Figures 4C, D**). Collectively, these data demonstrated rapid induction of IFN-γ as a main *in vivo* characteristic of activated lung T_RM_ cells, which might underlie the ability of these cells to promote an antiviral ISG program in the lung that occurs earlier than during primary response.

### IFN-γ Is a Major Mediator of the Antiviral Action of Lung CD8+ T_RM_ Cells Through Inducing ISGs and Inhibiting Inflammatory Cytokine Production

**Figure 5:**
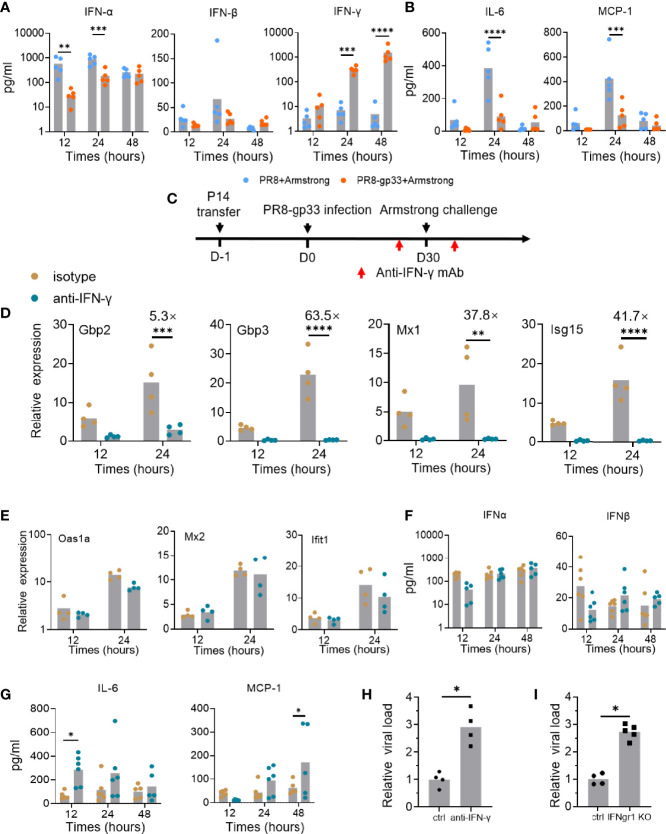
IFN-γ plays important roles in mediating the protective activity of lung CD8+ T_RM_ *via* contribution to induction of downstream ISGs and reduction of inflammation signals. **(A, B)** Naïve mice were subjected to P14 seeding and sequential infection with PR8-gp33/PR8 and LCMV Armstrong the same as described in **Figure 3A**. BAL was sampled with 1 ml of PBS at 12 h, 24 h, and 48 h after LCMV Armstrong challenge; the protein levels of IFN-γ and two type I interferons, and those of two representative proinflammatory cytokines, IL-6 and MCP-1, were assessed by cytometric bead array (CBA), respectively, shown in **(A, B)**. **(C–H)** Naïve mice were seeded with P14 cells, subjected to sublethal PR8-gp33 infection for memory induction, and then challenged with LCMV Armstrong while receiving an anti-IFN-γ IgG or an isotype-matched control IgG or intranasally at both 1 day before and 6 h after the challenge **(C)**. At various time points post challenge, lung and BAL were collected and analyzed respectively for mRNA expression of seven ISGs by quantitative RT-PCR **(D, E)**, and protein expression of IFN-α and IFN-β **(F)**, or two representative proinflammatory cytokines, IL-6 and MCP-1 by CBA assay **(G)**. **(H)** The lung viral loads were determined on day 5 after the LCMV Armstrong challenge. **(I)** IFN-γ receptor knockout (IFNgr1 KO) and wild-type mice (ctrl) were submitted to adoptive transferring of P14 cells, PR8-gp33 priming, and LCMV Armstrong challenge, following the schedule illustrated in **Figure 1D**. Shown are lung viral loads determined on day 5 after the LCMV Armstrong challenge. Data are representative of two or three independent experiments. *p < 0.05, **p < 0.01, ***p < 0.001, ****p < 0.0001; two-way ANOVA **(A, B, D–G)**, Mann–Whitney *U* test **(H, I)**.

Given the possibility that the level of secreted IFN protein might be regulated by post-transcriptional mechanism, we assessed the protein levels of IFN-γ and two type I interferons, IFN-α and IFN-β, in the BALs using cytometric bead array (CBA) assay. The PR8-gp33-primed group started to show enhanced level of BAL IFN-γ protein at 12 hpi as compared to the PR8-primed group; a larger difference between the two groups was observed at 24 hpi and 48 hpi as the BAL IFN-γ protein levels increased progressively in the PR8-gp33-primed group while being largely unchanged in their PR8-primed counterpart (**Figure 5A**). The inconsistency between sustained accumulation of BAL IFN-γ protein and decline of lung-localized IFN-γ mRNA from 24 hpi to 48 hpi in PR8-gp33-primed animal could be reconciled by delayed IFN-γ protein synthesis and/or that IFN-γ protein is more stable than its mRNA. Unlike IFN-γ, type I interferons showed unchanged or rather decreased protein level in the BAL of PR8-gp33-primed animal compared with PR8-primed animals (**Figure 5A**). These data strengthened the notion that lung CD8+ T_RM_ cells rapidly upregulate IFN-γ mRNA and consequently increase the amount of secreted IFN-γ upon activation.

IFNs are known to have paradoxical functions in the control and pathogenesis of viral infection (29). Besides the well-known viral inhibition activity, they might stimulate production of pro-inflammatory cytokines and their prolonged action could impair tissue recovery after viral clearance (30). Thus, we also assessed the temporal expression of two important inflammatory cytokines, IL-6 and MCP-1, in the BAL using CBA assay. In the absence of lung CD8+ T_RM_ cells (PR8-primed animal), both cytokines were lowly expressed at 12 hpi and then underwent a sharp rise to reach peak level at 24 hpi, thereafter declining to low level at 48 hpi. Under the action of lung CD8+ T_RM_ cells (PR8-gp33-primeed animals), the high induction of both cytokines at 24 hpi was pronouncedly suppressed (**Figure 5B**). These data suggested that activated lung CD8+ T_RM_ cells may facilitate lung recovery after infection by acting to restrain the elevation of proinflammatory cytokines.

As all the above data lend support for the notion that activated lung CD8+ T_RM_ cells rapidly secrete IFN-γ to induce timely expression of antiviral ISGs in the lung for control of viral infection, we sought to conduct a direct examination. To this end, we adopted a modified version of the second infection model, wherein an IFN-γ-targeting neutralization antibody or an isotype matched control antibody was intranasally administered both on 1 day before and at 6 h after the LCMV Armstrong challenge (**Figure 5C**). The antibody-mediated blockade of IFN-γ had no detectable effect on the number and frequency of P14 cells in the BAL fluid and their surface marker expression (**Figures S3A, B**), but it nearly completely abolished the transcriptional induction of four of the seven representative ISGs examined, namely, Gbp2, Gbp3, Mx1, and Isg15 (**Figure 5D**). For the other three ISGs, including Oas1a, Mx2 and Ifit1, their expression was unaffected by IFN-γ neutralization, indicative of them being possibly induced by other IFNs (**Figure 5E**). Indeed, the BAL levels of IFN-α and IFN-β proteins, as assessed by CBA assay, showed no significant changes upon IFN-γ blockade; thus, they and/or other type I IFNs may account for the IFN-γ-independent ISG induction (**Figure 5F**). Our CBA assessments also found that the expression of both IL-6 and MCP-1, two cytokines suppressed in the presence of activated lung T_RM_, was substantially elevated upon IFN-γ blockade, though the time point for most significant upregulation varied (**Figure 5G**). These results solidified the notion that IFN-γ facilitates the early action of lung T_RM_ in sculpting a potent tissue-wide ISGs program while keeping the inflammatory response under control to minimize collateral tissue damages. They also surprisingly revealed that part of this ISG expression program is independent of IFN-γ, suggesting the potential involvement of type I IFNs.

Finally, we examined the IFN-γ dependence of lung T_RM_ cells in protection against viral infection. The IFN-γ signaling was blocked in the second infection model using either IFN-γ neutralization antibody as described above, or IFN-γ receptor 1 (Ifngr1) knockout mice. In both contexts, the absence of IFN-γ signaling resulted in a significant, nearly 3-fold increase in lung viral load of LCMV Armstrong, thus validating the mediating role of IFN-γ in the antiviral activity of lung CD8+ T_RM_ cells (**Figures 5H, I**).

## Discussion

Among the three major subsets of memory CD8+ T cells, T_RM_ cells were lastly identified and have attracted extensive research in recent years. Despite sharing a core gene expression signature, T_RM_ cells derived from different tissues distinguish from each other in phenotype, homing property, and expression of distinct genes, reflecting adaptation to its habitat. Seminal studies of skin and FRT CD8+ T_RM_ cells demonstrated a paradigm for the antiviral action of T_RM_, in which these cells mainly serve a sentinel role through secreting IFN-γ and other cytokines to establish a tissue-wide antiviral state and recruit circulating immune cells. There has been limited evidence supporting the applicability of such paradigm to lung T_RM_ cells, the majority of which came from *ex vivo* analysis of isolated mouse lung T_RM_ cells. In this study, we explored the *in vivo* action of lung CD8+ T_RM_ in the early phase of viral infection, which represents a critical stage in the battle between memory immunity and respiratory virus infection. This exploration was facilitated by our establishment of two sequential infection models, allowing us to demonstrate the importance of lung CD8+ T_RM_ cells in memory response-mediated cross-protection against infections of H7N9 influenza virus as well as model virus. By simultaneously interrogating the dynamics of transcriptional landscapes of activated lung T_RM_ cells versus the infected whole lung conditioned or nonconditioned by these cells, we developed the notion that the very early response of lung T_RM_ cells upon antigen re-encountering was centered on rapid upregulation of IFN-γ, which resulted in an early wave of ISG induction, accompanied by reduction of proinflammatory cytokines. Subsequently, we could provide experimental evidence substantiating this notion while also implicating the existence of additional mechanism(s) besides IFN-γ contributing to the ability of lung CD8+ T_RM_ cells to induce ISGs and inhibit virus replications. Together, our study delineated the *in vivo* dynamic response of lung CD8+ T_RM_ cells upon activation, revealing a prompt action underscored by rapid IFN-γ production for triggering an antiviral state in the lung.

It should be appreciated that the side-by-side comparison of temporal gene expression pattern of lung CD8+ T_RM_ cells versus whole lung tissues is extremely important to differentiate the contribution of lung CD8+ T_RM_ cells and surrounding tissues to the induction of IFN-γ and downstream ISGs. Lung CD8+ T_RM_ cells as the major early provider of IFN-γ was evidenced by that, for the induction of IFN-γ and even more obviously for the induction of a subset of ISGs, these cells precede the whole lung. On the other hand, the lung features more highly induced ISGs, consistent with a scenario in which IFN-γ, secreted by lung CD8+ T_RM_ cells, acts in a paracrine manner on the surrounding or even distant lung cells to stimulate ISG expression. The relatively narrow spectrum of ISGs in lung CD8+ T_RM_ cells might be attributed to the cross-talk between IFN-γ signaling and/or interaction between these cells and antigen-presenting cells as suggested by a recent study (30).

The comparison of microarray data of whole lungs conditioned or nonconditioned by CD8+ T_RM_ cells further indicated that IFN-γ, but not IFN-α/β, is preferentially upregulated by lung CD8+ T_RM_ cells and may account for an early induction of a broad ISG response in the lung.

Our results also suggested that IFN-γ cannot be the only molecule that lung CD8+ T_RM_ cells utilize to exert its antiviral function. Based on the observation that the induction of three ISGs, namely, Oas1a, Mx2, and Ifit1, was largely unaffected by IFN-γ neutralization, we speculated that type I interferon might also contribute to the antiviral activity of lung CD8+ T_RM_ cells *via* induction of a portion of antiviral ISGs. In line with this speculation, a broad induction of type I interferon was observed in activated lung CD8+ T_RM_ cells at specific late time point(s) after IFN-γ upregulation. Furthermore, we could detect expression of IFN-α and IFN-β proteins in the BAL at 12–48 hpi that were independent of IFN-γ.

Another important impact of lung CD8+ T_RM_ cells on early memory response was reducing levels of inflammatory cytokines. This effect might be the consequence of reduced viral replication or reflective of biological activity of IFN-γ. Despite signaling through the same STAT1 transcription factor and consequently sharing many downstream ISG targets, IFN-γ and IFN-α/β recognized distinct surface receptors engaging different JAK platform, resulting in different kinetics of ISG induction and non-overlapping modulation on a subset of IFN-specific ISGs. In comparison to IFN-α/β, IFN-γ directs a weaker induction for many of the shared ISG targets in addition to having fewer unique downstream targets. On the other hand, titration studies revealed that cells were more sensitive to IFN-γ than IFN-α, as justified by lower units for achieving the same degree of STAT1 phosphorylation (31). Thus, activated lung CD8+ T_RM_ cells might strategically regulate the induction of IFN-γ and type I interferon in a temporal manner, thus allowing them to leverage different properties of the two IFN types at different phase of infection to inhibit the virus while limiting the inflammation-caused lung damage. Such programmed utilization of IFN-γ and type I interferon could render the resulting antiviral immunity more favorable than an immunity dominated by type I interferon(s), as seen with the primary response or a recall response where the input of lung T_RM_ cells is absent. As our microarray analyses failed to reveal the expression of type III interferons, we cannot exclude the possibility that these interferons, which have been shown to possess antiviral activity (32, 33), may also participate in the ISG expression program induced by lung CD8+ TRM cells. Such possibility is certainly worthy of future investigation.

One caveat of our studies is that we were not able to differentiate the contribution of airway versus lung parenchymal T_RM_ cells to the elevated lung IFN-γ expression and the protection against secondary infection. Previous *ex vivo* characterization of airway T_RM_ cells showed their superiority in IFN-γ production with limited cytolytic activity compared to their parenchymal counterparts, leading to the postulation that airway and lung parenchymal T_RM_ cells divide the labor in the memory immune defense against virus invasion. We agree with this division-of-labor concept. Nevertheless, our results highlight the essentiality of lung CD8+ T_RM_ cells for cross-protective memory immunity against respiratory viral infection, thereby pointing to the engagement of lung CD8+ T_RM_ cells as a major prerequisite of a broad-spectrum influenza vaccine. Indeed, a real example was presented in the study, that is, the utilization of normally harmless H9N2 virus as a vaccine against H7N9 virus. Equally importantly, we extend the perspective that there exist intrinsic mechanisms governing lung T_RM_ to rapidly respond to antigen re-exposure. There is reason to believe that future elucidation of the cellular and molecular basis for rapid activation of lung T_RM_ cells would facilitate the development of vaccination or immunotherapeutic strategies aimed at optimizing the beneficial effects of these fast-acting cells.

## Data Availability Statement

The datasets presented in this study can be found in online repositories. The names of the repository/repositories and accession number(s) can be found in the article/**Supplementary Material**.

## Ethics Statement

The animal study was reviewed and approved by Shanghai Public Health Clinical Center Laboratory Animal Welfare & Ethics Committee.

## Author Contributions

JX and XZ conceived this study. JX and XZ designed the experiments. LJ, LL, CuZ, LZ, and QH performed the experiments. LJ and LL analyzed the data. ZL and MZ provided the virus and reagents. MZ and LZ prepared the virus. LJ prepared the manuscript. JX, XZ, ZL, and ChZ revised the paper. All authors contributed to the article and approved the submitted version.

## Funding

This work was supported by the National Natural Science Foundation of China (81672018, 82071788, and 81771704).

## Conflict of Interest

The authors declare that the research was conducted in the absence of any commercial or financial relationships that could be construed as a potential conflict of interest.

## Publisher’s Note

All claims expressed in this article are solely those of the authors and do not necessarily represent those of their affiliated organizations, or those of the publisher, the editors and the reviewers. Any product that may be evaluated in this article, or claim that may be made by its manufacturer, is not guaranteed or endorsed by the publisher.
