# Supplementary material for: Prompt Antiviral Action of Pulmonary CD8+ TRM Cells Is Mediated by Rapid IFN-γ Induction and Its Downstream ISGs in the Lung
Source: Front Immunol. 2022 Feb 22;13:839455. doi: 10.3389/fimmu.2022.839455 (PMC8920550; doi:10.3389/fimmu.2022.839455)
Supplement: Supplementary file 1 [file DataSheet_1.docx]

**Supplementary information**

**Supplementary figures:**


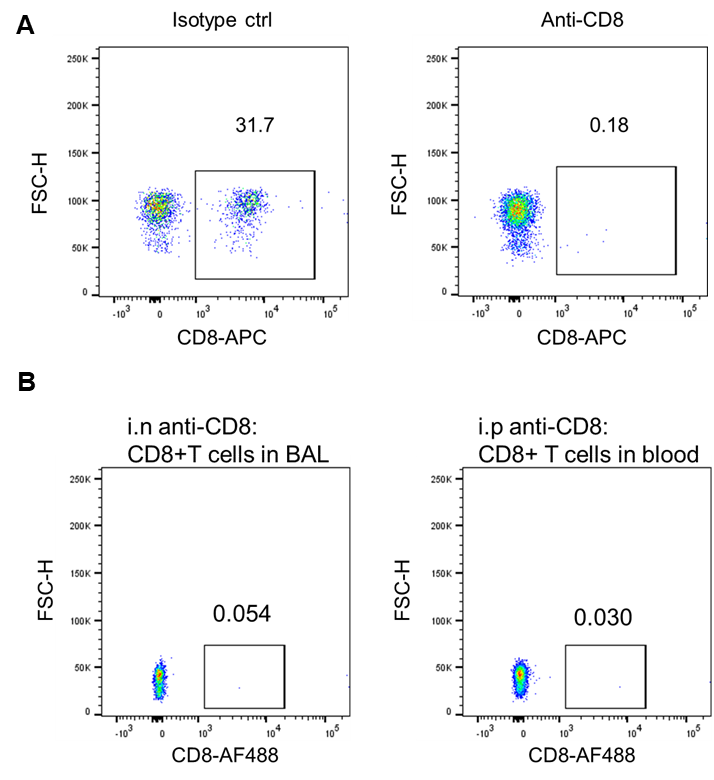


**Figure S1: FACS data supporting the efficacy of antibody-mediated depletion of the CD8+ T cells.** (**A**) The example of flow plots demonstrating the CD8+ T cell depletion strategy using anti-CD8 antibody related to Fig 2A. (**B**) Flow cytometry showing CD8+ T cell depletion related to Fig 2G.

**
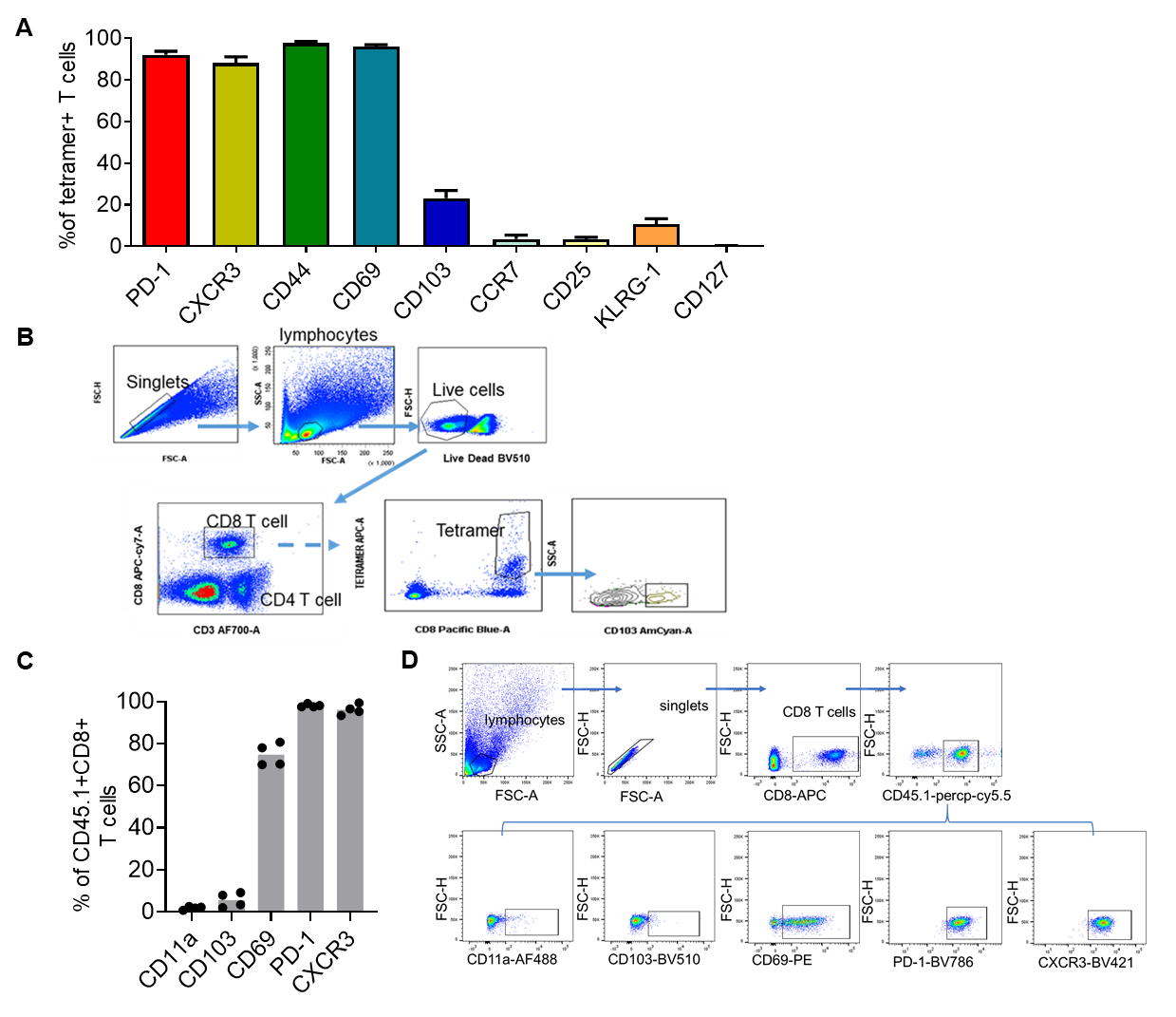
**

**Figure S2: Phenotypic characterization of airway T_RM_ cells.** (**A**-**B**) Expression of the indicated surface markers on NP366-374 tetramer+ TRM cells in the H9N2 primed BAL fluid, assayed by flow cytometry at 30 days after the priming. (**C**-**D**) Surface marker profiling of PR8-gp33-primed, adoptively transferred P14 cells by flow cytometry at 30 days after the priming. Related to Fig. 1D.


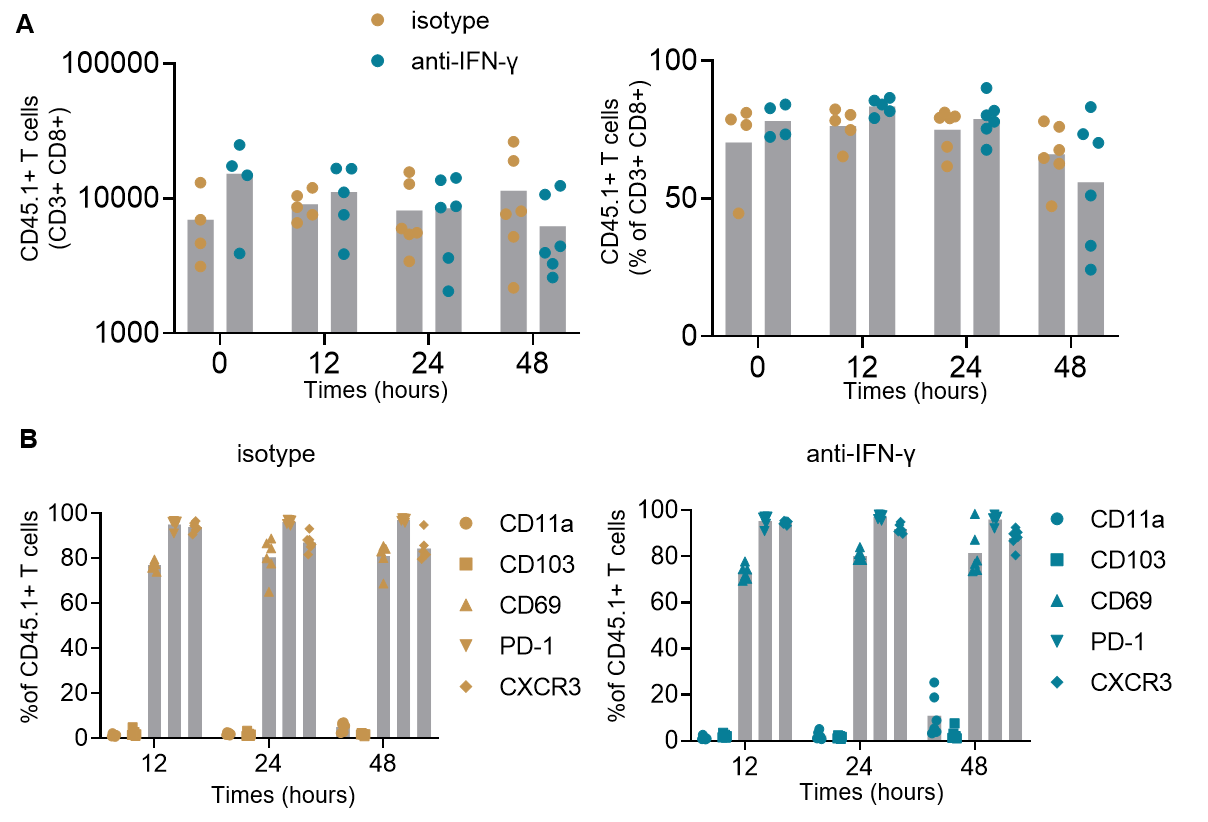


**Figure S3: Neutralization of IFN-γ did not change the number and phenotype of lung CD8+ T_RM_ cells.** The experimental setting was described in Fig. 4C. Flow cytometry was used for determining the effect of administration of an IFN-γ-targeting antibody (anti-IFN-γ) versus an isotype-matched control antibody (isotype) on the number and frequency of T_RM_ cells in the BAL fluid (**A**) and their surface marker expression (**B**). in (**B**), the data for control antibody-treated group (left panel) and anti-IFN-γ antibody-treated group (right panel) were separately displayed in left and right panel. Data are representative of two independent experiments. Related to Fig. 4.

**Supplementary table:**

**Primers used for RT-PCR**

| Gene | primer |
| --- | --- |
| Actinβ F  Actinβ R | 5’-GTGACGTTGACATCCGTAAAGA-3’  5’-GCCGGACTCATCGTACTCC-3’ |
| IFN-γ F  IFN-γ R | 5’-ACAGCAAGGCGAAAAAGGATG-3’  5’-TGGTGGACCACTCGGATGA-3’ |
| Mx1 F  Mx1 R | 5’-GGGGAGGAAATAGAGAAAATGAT-3’  5’-GTTTACAAAGGGCTTGCTTGCT-3’ |
| Mx2 F  Mx2 R | 5’-TTCACCAGGCTCCGAAAAGA-3’  5’-CAGCTCGTACAATTTCAGTGACC-3’ |
| Gbp2 F  Gbp2 R | 5’-AGTCAATGGGCCACGTCTAA-3’  5’-GCCTCACTCTCAATTGGCCT-3’ |
| Gbp3 F  Gbp3 R | 5’-CTGACAGTAAATCTGGAAGCCAT-3’  5’-CCGTCCTGCAAGACGATTCA-3’ |
| Oas1a F  Oas1a R | 5’-CAGCCTTTGATGTCCTGGGT-3’  5’-CCCAGCTTCTCCTTACACAGT-3’ |
| Isg15 F  Isg15 R | 5’-CAGCAATGGCCTGGGACCTAA-3’  5’-AGGGTAAGACCGTCCTGGAG-3’ |
| Ifit1 F  Ifit1 R | 5’-TTACAGCAACCATGGGAGAGAA-3’  5’-CTCACGTAGGCCAGGAGGTT-3’ |
| LCMV F | 5’-CATTCACCTGGACTTTGTCAGACTC-3’ |
| LCMV R | 5’-GCAACTGCTGTGTTCCCGAAAC-3’ |
